# Supplementary figures and images for: Two cold shock domain containing proteins trigger the development of infectious Trypanosoma brucei
Source: PLoS Pathog. 2023 Jun 5;19(6):e1011438. doi: 10.1371/journal.ppat.1011438 (PMC10270622; doi:10.1371/journal.ppat.1011438)

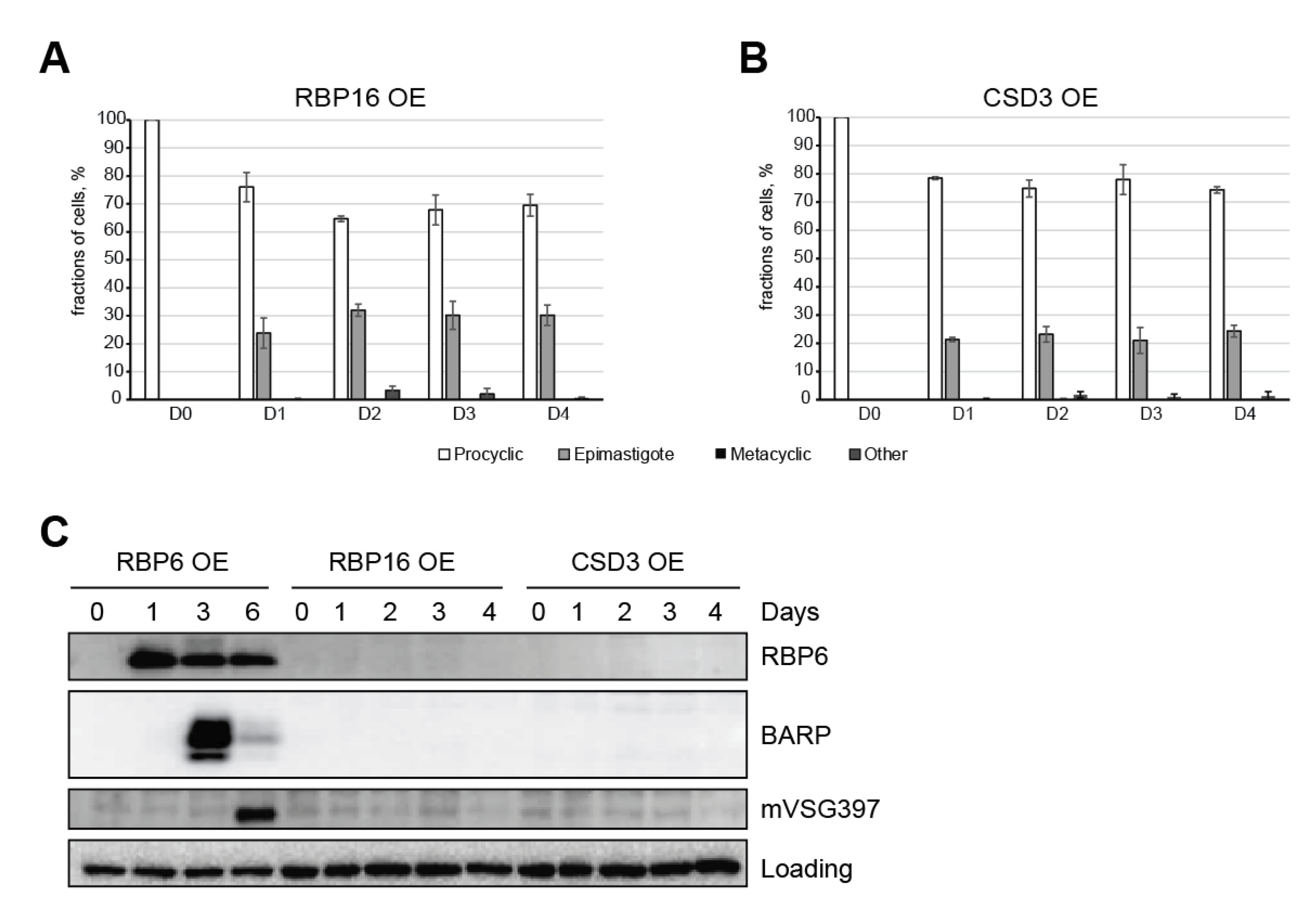

Supplement: S1 Fig — Induced RBP16 (A) and CSD3 (B) were scored for procyclic, epimastigotes, and metacyclic parasites over a 4-day period. The various developmental stages were scored as previously described (Kolev NG, Ramey-Butler K, Cross GA, Ullu E, Tschudi C. Developmental progression to infectivity in Trypanosoma brucei triggered by an RNA-binding protein. Science. 2012; 338:1352–1353). Three independent biological replicates were performed and at least 100 cells were counted for each time point with means ± standard deviation (std). (C) Extracts from the induced RBP16 and CSD3 overexpression cell lines were probed for RBP6, BARP, and mVSG397 by Western blot. The expression profile was compared to that of the RBP6 overexpression cell line. (TIF) [file ppat.1011438.s001.tif]

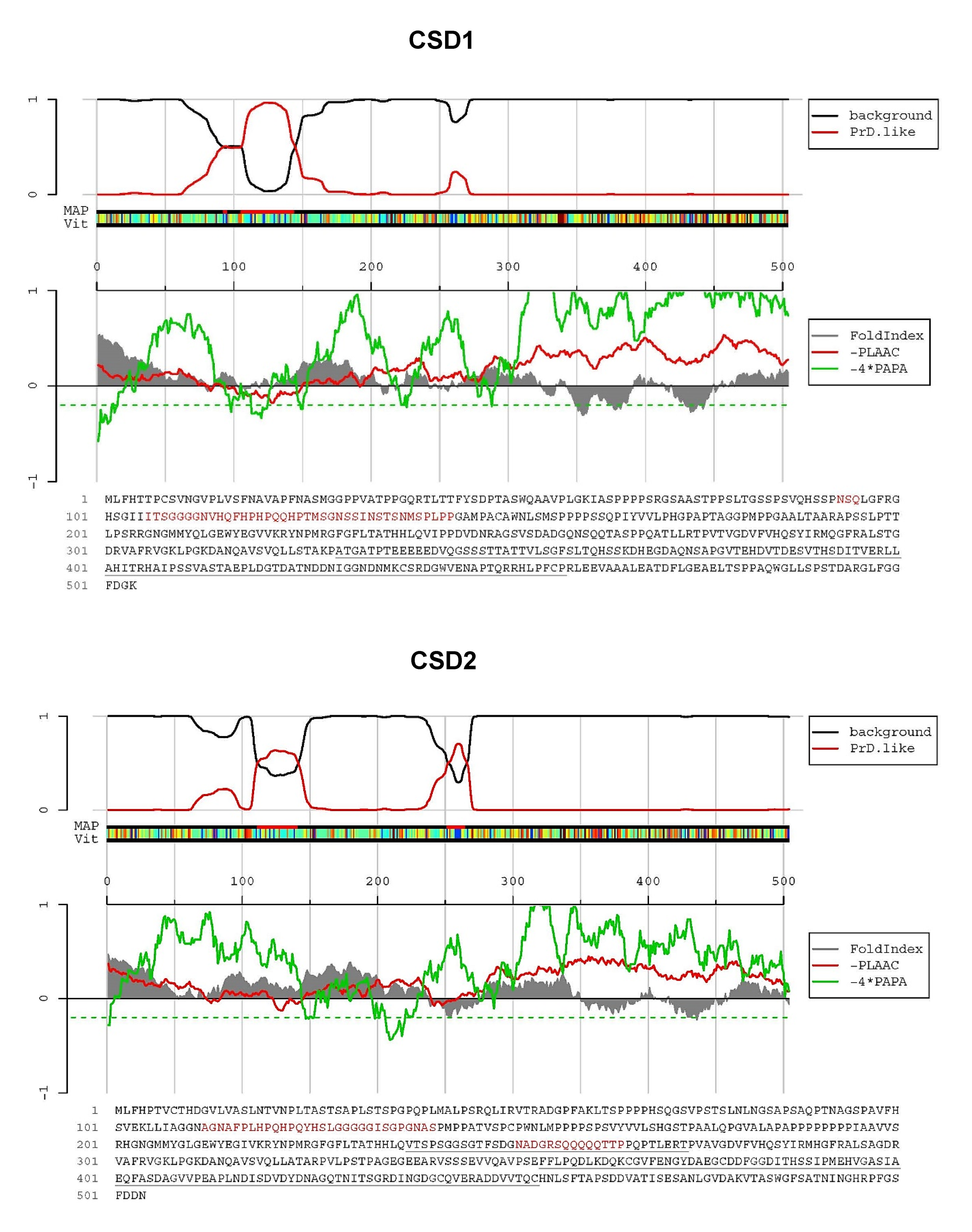

Supplement: S2 Fig — We scanned CSD1 and CSD2 amino acids using the Prion-Like Amino Acid Composition (PLAAC) program [33] and the prion-like domains are highlighted with red amino acids. (TIF) [file ppat.1011438.s002.tif]

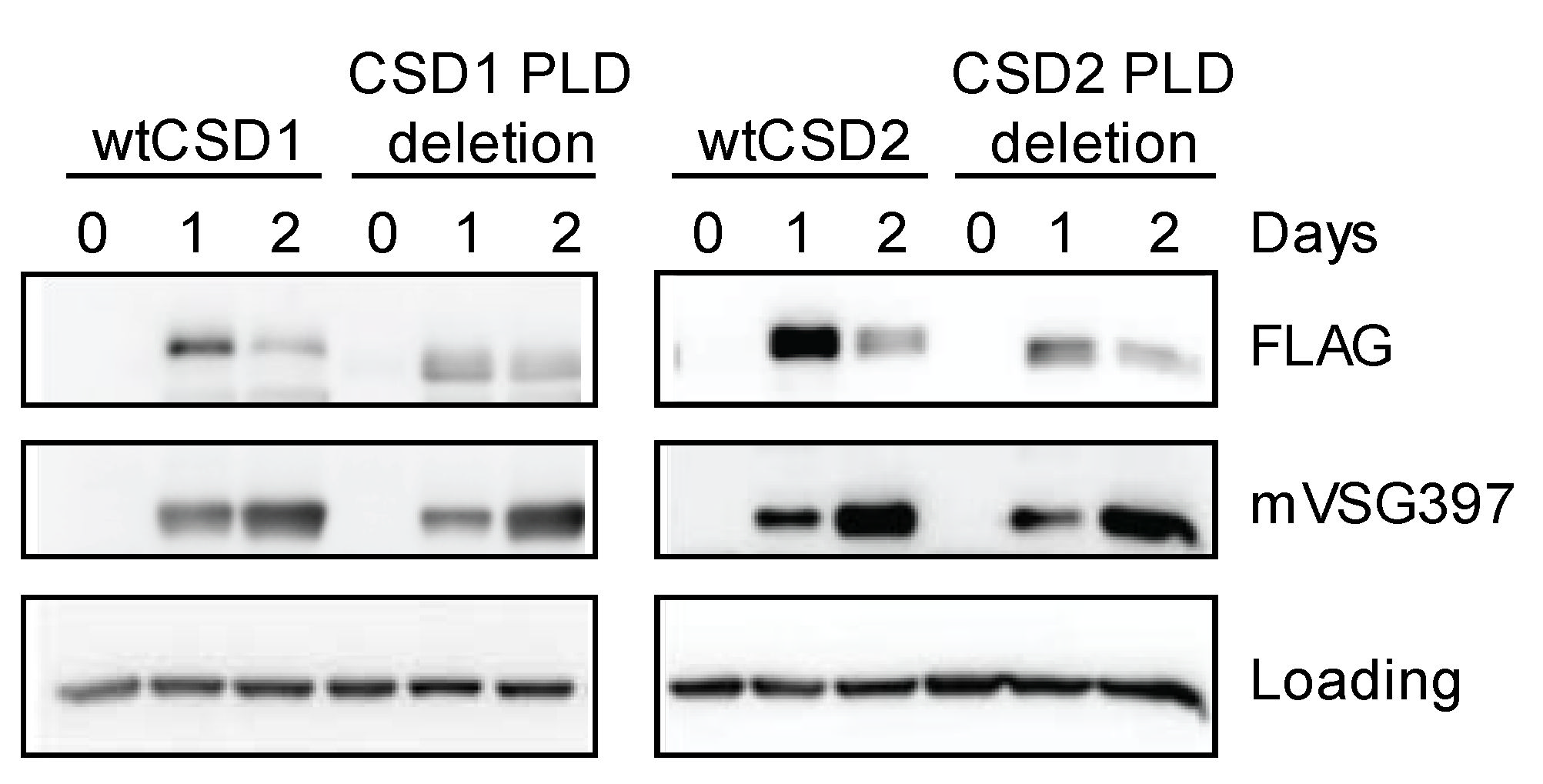

Supplement: S3 Fig — Procyclic parasites that ectopically expressed CSD1-3xFLAG or CSD2-3xFLAG with the PLD deleted were harvested in a 2-day period for Western blot analysis. The loss of the PLD domain in both cell lines did not affect mVSG397 expression. wtCSD1 and wtCSD2 serves as the positive control. (TIF) [file ppat.1011438.s003.tif]

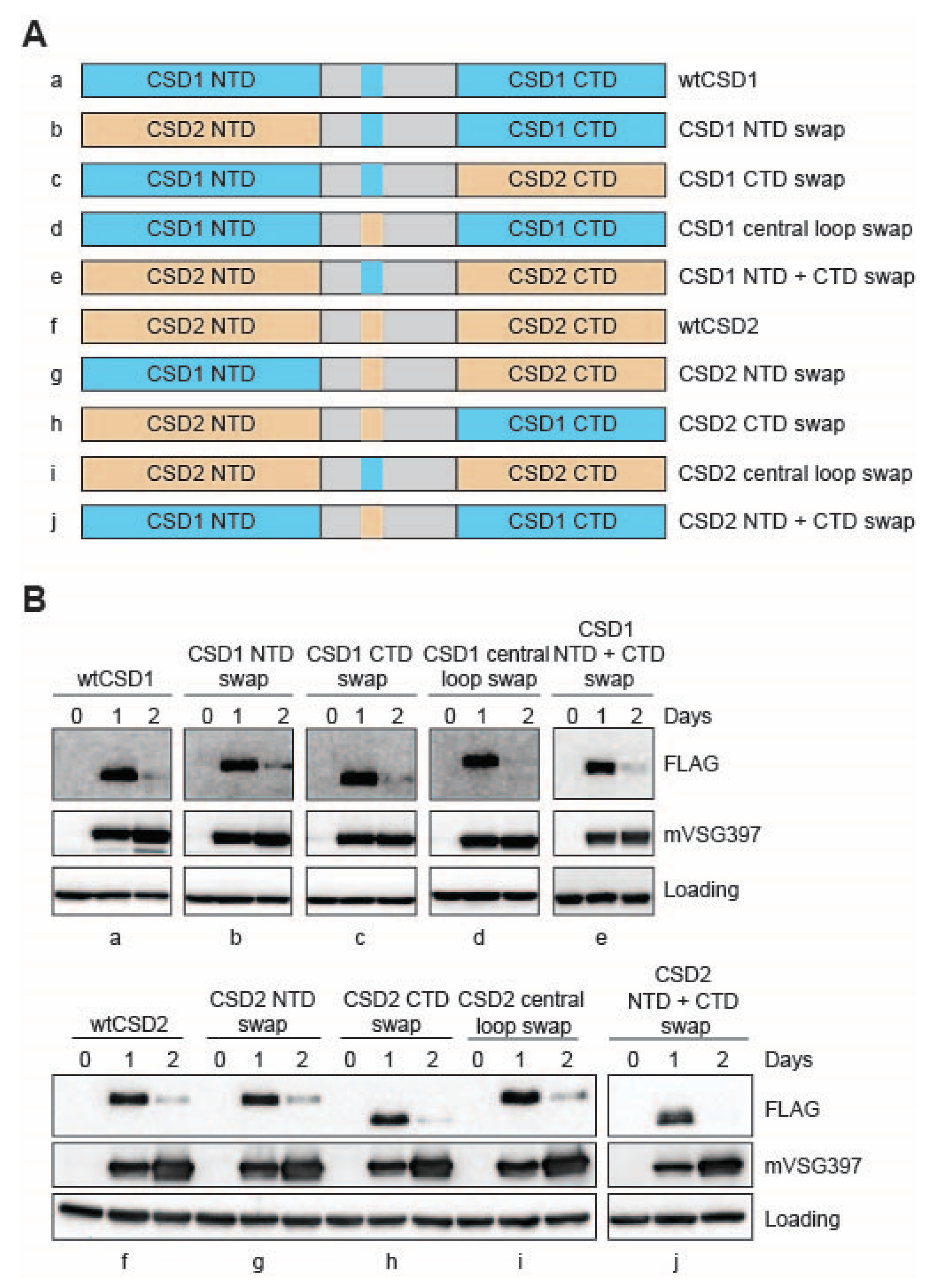

Supplement: S4 Fig — (A) Illustrations of the various combinations of NTDs, non-conserved sequences in the CSDs, and CTDs that were used in the generation of chimeric CSD1 and CSD2 proteins. (B) Procyclic cells individually overexpressed one of the eight chimeric proteins. None of the eight cell lines were impaired in their ability to express mVSG397. wtCSD1 or wtCSD2 were used as positive controls. (TIF) [file ppat.1011438.s004.tif]

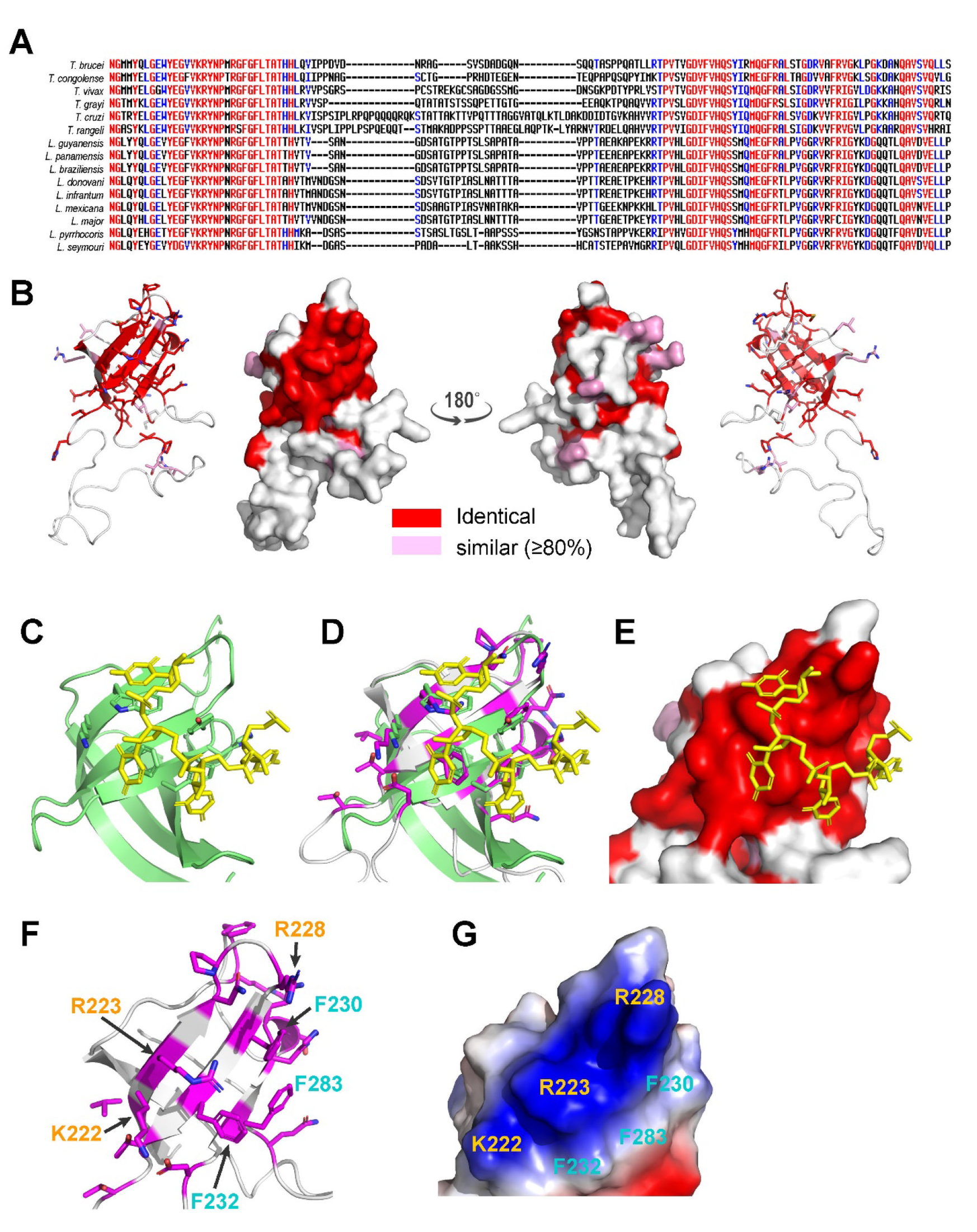

Supplement: S5 Fig — (A) Sequence alignment of the CSD of CSD1 with homologs from various kinetoplastid species. (B) Conservation plot of CSD1 based on the alignment in (A). (C) Ribbon diagram of YB-1 (green) in complex with its RNA target (yellow; PDB code: 5YTS). (D) Superposition of the YB-1/RNA complex structure on top of the homologous model of CSD1. Highly conserved residues in CDS1 are shown as magenta sticks. (E) Overlay of the RNA target of YB-1 on the conservation plot of CSD1 based on superposition in (D). (F) Ribbon diagram of CSD1 with all highly conserved residues highlighted in magenta. The two groups of positively charged and hydrophobic residues mutated in this study are labeled and colored in orange and cyan, respectively. (G) Electrostatic surface plot of CSD1 in the same orientation as in (F). (TIF) [file ppat.1011438.s005.tif]

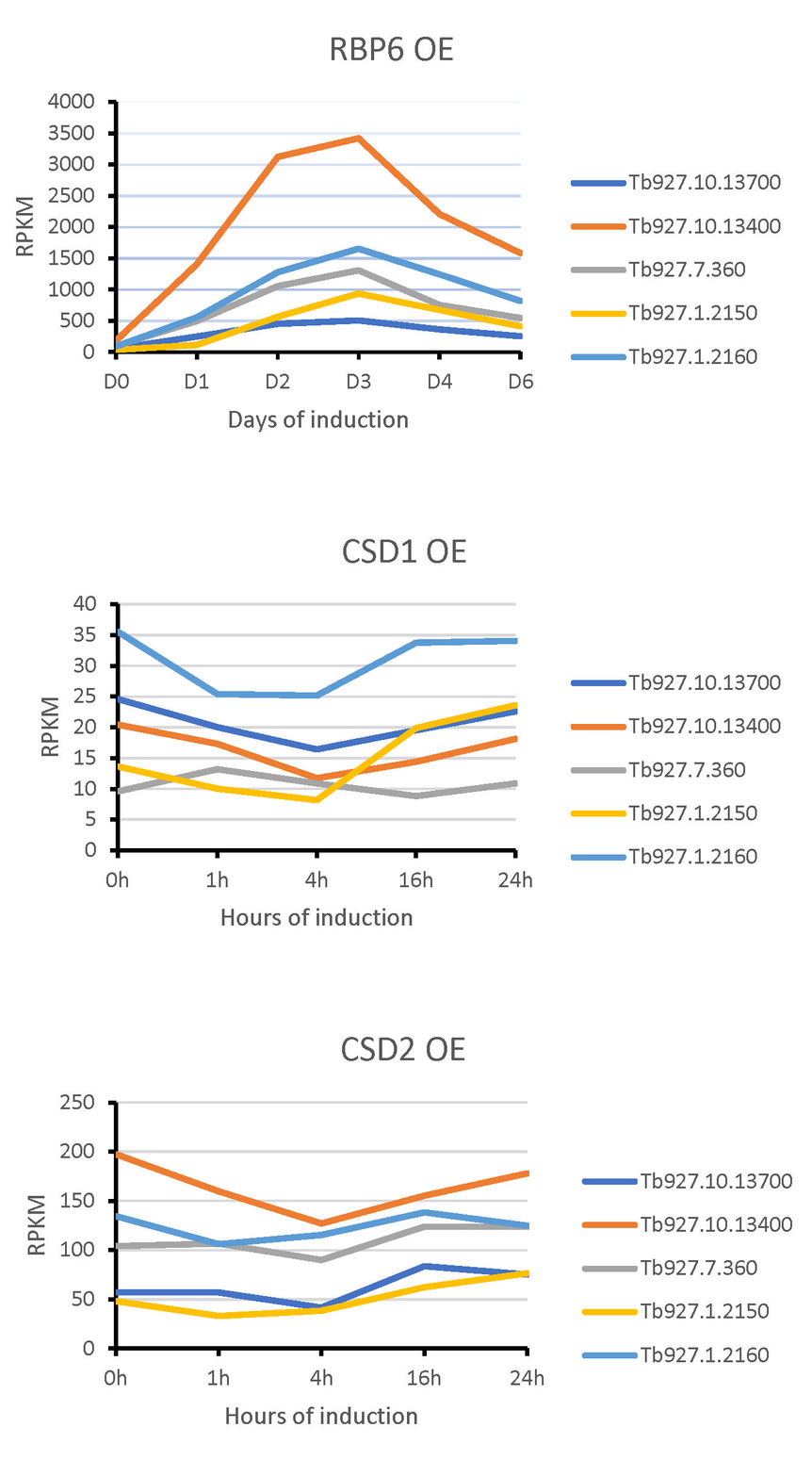

Supplement: S6 Fig — Single cell RNA-Seq of trypanosome development in the tsetse fly vector identified 5 epimastigote markers (Vigneron et al. PNAS 117, 2613–2621, 2020): phosphatidic acid phosphatase alpha, putative (Tb927.10.13700), phosphatidic acid phosphatase, putative (Tb927.10.13400), SGE1 (Tb927.7.360), small kinetoplastid calpain-related protein 1–4 (Tb927.1.2150), and small kinetoplastid calpain-related protein 1–5 (Tb927.1.2160). (TIF) [file ppat.1011438.s006.tif]

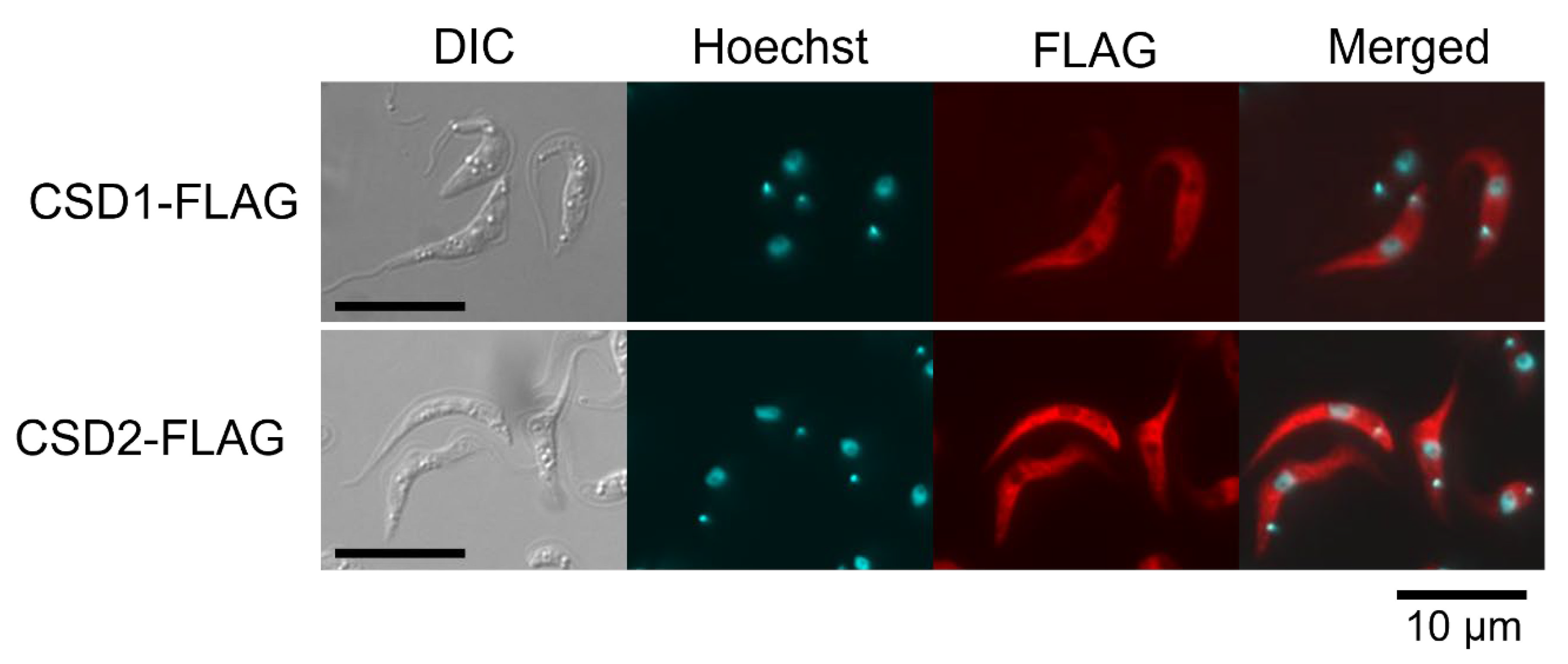

Supplement: S7 Fig — (TIF) [file ppat.1011438.s007.tif]

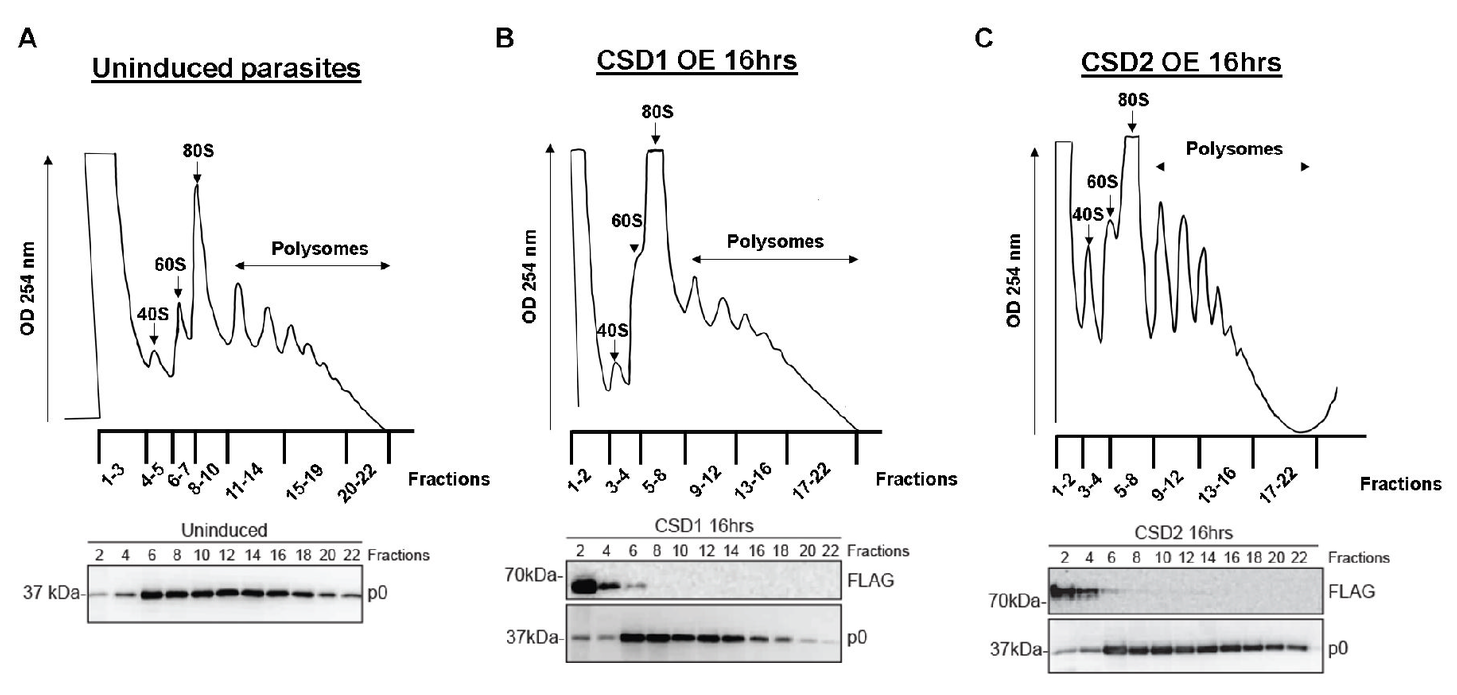

Supplement: S8 Fig — Lysates obtained from un-induced (A) and 16 h induced CSD1 (B) and CSD2 (C) overexpression cell lines were loaded on a linear 15–50% sucrose gradient and the absorbance was recorded at 254 nm. The positions of 40S, 60S, 80S and polysomes are indicated. The six pooled gradient fractions are: 1–2 (free RNA), 3–4 (40S), 5–8 (60S and 80S), 9–12 (light polysomes), 13–22 (heavy polysomes). The bottom panels show Western blots performed on each designated fraction for ribosomal p0 protein or for the 3xFLAG-tagged protein. (TIF) [file ppat.1011438.s008.tif]

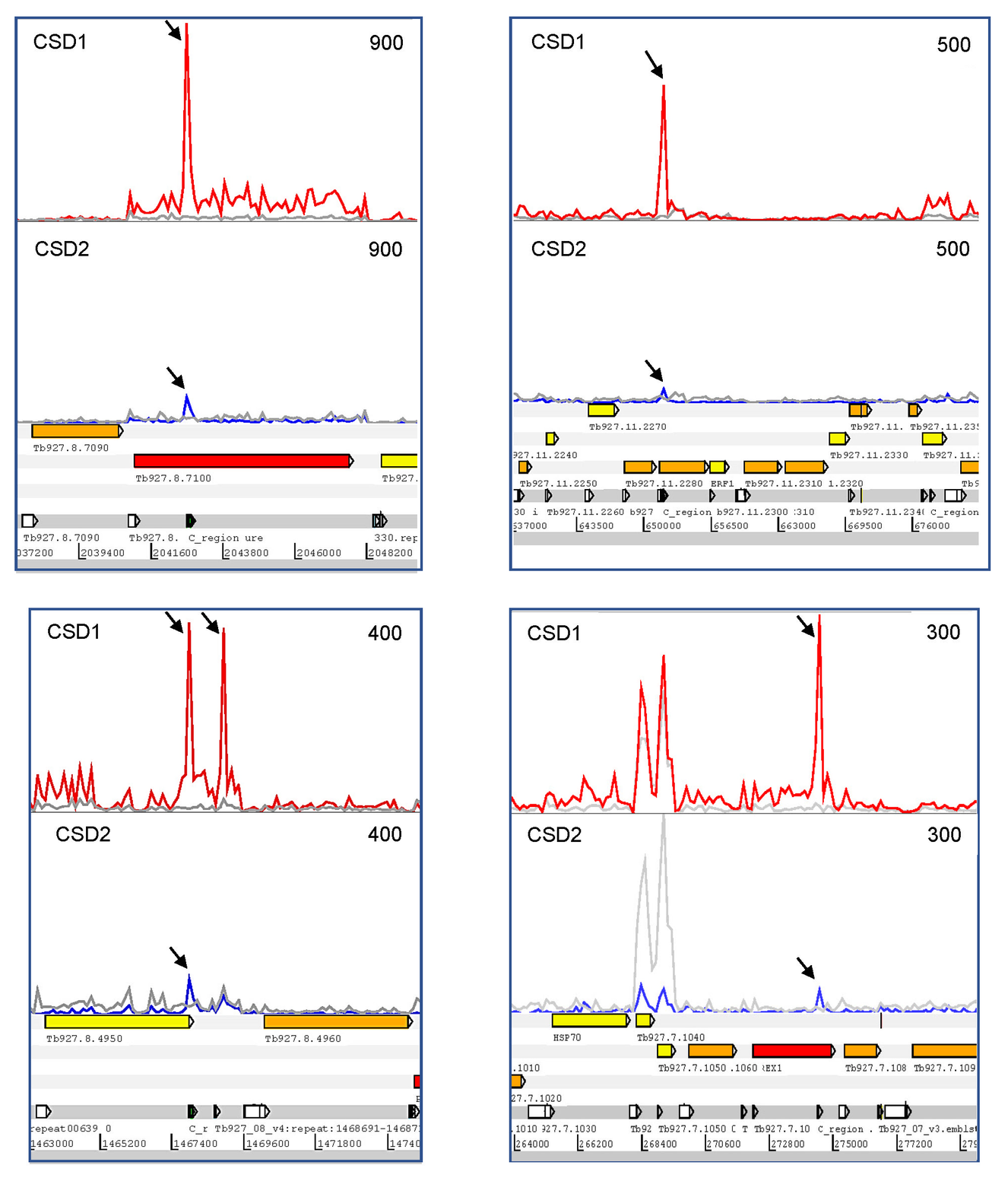

Supplement: S9 Fig — CSD1 (red line), CSD2 (blue line) and corresponding input controls (gray line) are shown. The number on the right indicates the scale of the plot area. Annotated peaks are pointed out by arrows. (TIF) [file ppat.1011438.s009.tif]
