# Supplementary material for: Two cold shock domain containing proteins trigger the development of infectious Trypanosoma brucei
Source: PLoS Pathog. 2023 Jun 5;19(6):e1011438. doi: 10.1371/journal.ppat.1011438 (PMC10270622; doi:10.1371/journal.ppat.1011438)
Supplement: Attachment — Submitted filename: Response to Reviewers.docx [file ppat.1011438.s015.docx]

*Christian Tschudi, Ph.D.*

*Department of Epidemiology of*

*Microbial Diseases*

*School of Public Health*

*BCMM 136C*

*295 Congress Avenue*

*New Haven, CT 06536-0812*

*Telephone: 203 785-7332*

*e-mail: christian.tschudi@yale.edu*

Dr. Keith R. Matthews	April 26^th^, 2023

Guest Editor

PLoS Pathogens

Dear Keith,

I am submitting a revised version of our manuscript entitled “Two cold shock domain containing proteins trigger the development of infectious *Trypanosoma brucei*” for consideration for publication in PLoS Pathogens. We addressed the comments of the three reviewers as outlined in detail below and hope that the manuscript is now acceptable for publication.

Sincerely yours,

Christian Tschudi, Ph.D.

John Rodman Paul Professor of Epidemiology

Yale School of Public Health

**Response to Reviewer comments**

***Part II – Major Issues: Key Experiments Required for Acceptance*** *Please use this section to detail the key new experiments or modifications of existing experiments that should be absolutely required to validate study conclusions.*

*Reviewer #1: Fig 2: The authors should also assess these cells using flow cytometry to determine cell cycle phase, since DAPI staining and microscopy, without further quantification, does not reveal nuclear DNA content.*

We added the following sentence “There was no kinetoplast elongation in the 1K1N cells, excluding the possibility that they have entered S phase (Siegel *et al* 2008).”

*Some data are difficult to assess without statistical analysis. In Fig 7B, for example, are these cohort enrichments significant (unclear what the axis represents)? It’s also unclear whether CSD1 binds the sequence shown in Fig 7C. What’s the frequency of occurrence and p-value associated with this putative ‘CSD1-binding motif’ for example when the set of 233 high-confidence targets are compared to a control set of sequences using the MEME suite?*

Fig 7B, now Fig 7C, has been replaced with a GO analysis. The axis is now labeled and represents -log(p-value).

New text related to Fig 7C, now Fig 7B,“In order to address the RNA sequence specificity of the 308 CSD1 peaks, we used the motif analysis algorithm MEME to search for sequence motifs in a 30-nt window around the RBP binding sites. This analysis retrieved ANACAU in 301 peaks as the most significantly enriched sequence motif (e-value: 1.4^-327^)(Fig 7B). 12 transcripts had multiple peaks and thus, we identified 289 target transcripts for CSD1 (S4 Table).”

*Reviewer #2: • The difference in RNA-seq results between RBP6 and CSD1/2 overexpression are discussed. What is the composition of the population of each sample sequenced? do the differences in transcriptomes result from a lack of epimastigotes after CSD1/2 overexpression, that are present in the RBP6 overexpression sample? Are epimastigote markers unchanged in CSD samples but altered in RBP6, reflecting this?*

For CSD1 and CSD2 the composition for 24 hours induction is shown in Figs. 1A and 1B. For RBP6 the 1-6 days composition is in our published papers (Kolev *et al.*, Science 2000, Shi *et al.* MBP 2018).

We thank the reviewer for pointing out the epimastigote markers. We added the following text and supplementary figure S6 “Conversely, 5 epimastigotes markers, previously identified by single cell RNA-Seq of trypanosome development in the tsetse fly vector, were up-regulated around day 3 in the RBP6 induction, but did not significantly change in transcript abundance in the CSD1 or CSD2 overexpression (S6 Fig), providing further evidence that CSD1/2 overexpression did not result in the production of epimastigotes.

*What is the composition of life cycle cell types in the RBP6 over expression samples when RNA was isolated? Was this performed in this study, or are comparisons made to previously published work?*

The comparison was made to previously published RNA-Seq data (Shi *et al*, MBP 2018). A reference was added.

*Authors claim “more pronounced BSF-like transcriptome” in CSD1/2 overexpression relative to RBP6, but is this due to the nature of the mixed populations? And less so to the nature of the resulting metacyclics themselves? Does the former contain a greater proportion of metacyclics at the shown timepoint than the latter, therefore skewing results.*

These are very valid points and thus, we deleted the statement “more pronounced BSF-like transcriptome”.

*seCLIP analysis details*

*what were the normalisation and peak identification steps? Was custom code used? Peaks are defined by an “arbitrary cutoff of 100” – 100 what exactly?*

No custom code was used. As stated in the text on lines 348-350 of the original manuscript “To identify seCLIP peaks, we manually inspected the aligned data and considered crosslinked sites with a read count of at least fifty and at least five-fold above the input control.” We removed the arbitrary cutoff.

*Why was normalisation done to previous RNA-seq and not input controls? Shouldn’t inputs from the same sample account for RNA abundance differences?*

The input is a control for the efficiency of the crosslinking, but is not an accurate representation of transcript abundance, due to the very limited coverage of average and low abundance transcripts in comparison to RNA-seq data. Thus, we used the uninduced RNA-Seq data of the CSD1 OE data set.

*What exactly were these previous RNA-seq samples used for normalisation (4, 16 or 24hr time points? Combined?)?*

We used the uninduced RNA-Seq data of the CSD1 OE data set*.*

*Can the authors please clarify if the resulting sample composition was similar between seCLIP and RNA-seq? Especially since CSD1 seCLIP sample is a combination of multiple time points across mixed populations. RNA abundance could be different between these experiments and so this may not be an appropriate control to normalise the peaks.*

See above.

*Input samples were generated, can the authors please clarify how they were used in analysis?*

Size-matched input controls taken from UV crosslinked samples, were used as background read counts, i.e. annotated seCLIP peaks had to be at least five -fold above the input control. This is stated in the original manuscript on lines 349 and 350.

*The data is not shown other than a list of peaks in the supplementary table. It would be useful to see example plots of the mapped peaks, compared to the appropriate controls. And to show difference between CSD1 and CSD2, which led to claims that peaks were likely missing in the latter and that each protein in fact has identical targets, even though these aren’t identified for CSD2.*

Four example plots are now shown in S9 Fig.

*Sup table has 308 peaks, why were peaks some removed to leave 289?*

We apologize for not explaining this clearly. New text “In order to address the RNA sequence specificity of the 308 CSD1 peaks, we used the motif analysis algorithm MEME to search for sequence motifs in a 30-nt window around the RBP binding sites. This analysis retrieved ANACAU in 301 peaks as the most significantly enriched sequence motif (e-value: 1.4^-327^)(Fig 7B). 12 transcripts had multiple peaks and thus, we identified 289 target transcripts for CSD1 (S4 Table).”

*Reviewer #3: The only major concern I have is presentational.*

*The results sections describes the numbers of transcripts that change on over expression, the overlap with those that change on RBP6 over expression. This is a little turgid and I wonder whether the dat would be better presented in a table?*

The overlap data and transcripts unique to CSD1 or CSD2 are now presented in S1 and S2 Tables.

***Part III – Minor Issues: Editorial and Data Presentation Modifications*** *Please use this section for editorial suggestions as well as relatively minor modifications of existing data that would enhance clarity.*

*Reviewer #1:*

*Several legends have insufficient detail: For example, are the replicates technical or biological and what do the error bars represent in fig 1A-B?*

Replicates are biological and error bars represent standard deviation. The Figure legend was modified.

*Which antibodies were used to detect the four proteins in fig 1D?*

The following was added in Materials and Methods “The following antibodies were used: for RBP6 a rabbit antiserum raised against the peptide QPYHPFATTEPQARLYPYHYC, for mVSG 397 a rabbit serum against the peptide CSDDAATYTSGSIAGTHALG, Dr. Isabel Roditi provided the anti-BARP antibody, and for the loading control against elongation factor 1-alpha (EF-1α, clone CBP-KK1 from Sigma- Aldrich).”

*Are the replicates technical or biological in Fig 2?*

Biological. The figure legend was modified.

*How was Fig 7C generated?*

MEME. Added to figure legend.

*In the abstract and author summary etc, should ‘ANACAT’ be ‘ANACAU’?*

Correct. Changes were made throughout the manuscript.

*Fig 2B is cited before 2A, and Fig 3B is cited before 3A.*

Figures were switched.

*The authors could consider shortening the ‘transcriptome’ sections from line 254-332.*

The transcriptome sections, as suggested, were shortened and the deleted information is now included in S1 and S2 Tables.

*Reviewer #2:*

*The authors refer to “purified metacyclics” in reference to fig 2B and RNA-sew results. How were these purified? There are no methods to refer to.*

A reference was added in the text and methods section.

*Details of RNA-seq analysis should be added. How was differential expression analysis performed? What is the significant and fold-change threshold used to select up and down regulated genes?*

The following text was added in Materials and Methods “Briefly, the reads of 75 nt were mapped to the *T. brucei* 11 megabase chromosomes (GeneDB version 5) using the Lasergene 17.1 software package from DNASTAR. The SeqMan NGen layout algorithm by DNASTAR is based on a local match percentage and the match percentage threshold has to be met in each overlapping window of 50 bases. We used a minimum aligned length of 70 nt and allowed a maximum of two mismatches. Reads with multiple matches in the genome were distributed randomly between the identical sequences, and the genes were counted as separate. For normalization, we used RPKM (reads assigned per kilobase of target per million mapped reads). For statistical analyses of differentialy expressed genes (DEGs), we used DESeq2 included in the Lasergene package. Adjusted p-value (false discovery rate, FDR) was calculated using the Benjamini-Hochberg method for multi-comparison. DEGs were filtered for fold-change >2 and adjusted p-value <0.05.”

*Cell types in fig 1 – is this based on morphology or marker expression?*

For Figure 1A and B cell types are based on morphology and kinetoplast repositioning, which was verified by marker expression in Figure 1D. The Figure legend was modified to “The induced CSD1 and CSD2 overexpression (OE) cell lines were monitored daily by light microscopy up to 4 days and the presence of various developmental trypanosome forms was based on morphology after DNA staining.”

*In cell counting figures (1,S1, 2b) how many cells were counted? Example images used to score each cell type would also be useful*

At least 100 cells were counted. The reference Kolev *et al*. Science 2010, was added for example images. The figure legends were modified accordingly.

*Scale bar needed in microscopy Fig S6*

A scale bar was added.

*Figure S1 - Images to show epimastigotes would be helpful, especially as BARP was not detected by western blot.*

Unfortunately, we do not have images. We added the reference Kolev *et al.* (2010) for representative pictures.

*Fig 3B is referred to before 3A in the results text, switch for clarity?*

The figures were switched.

*Lines 198-200. Authors claim all CSD mutants are expressed to similar levels, but CSD1 NTD deletions looks to be higher expressed. Although this probably does not affect the claims, as this mutant does not express mVSG. Authors could quantify the expression difference, or clarify in text.*

The text was changed to “expressed the truncated proteins at levels similar or higher”.

*What is the loading control used in western blots?*

Elongation factor 1-alpha (EF-1α). This was included in the legend of Fig. 1 and a reference was added.

*There is no figure legend for Figure S4*

Figure legend was added.

*VSG RNA mapping – was there a quality control step to remove multimapping reads to VSGs?*

We are aware that VSG genes share substantial sequence similarities, in particular towards the 3′ end of the gene. Thus, read coverage was visually inspected using the DNASTAR SeqMan NGen program, and only genes with a uniform read coverage were included in the data analysis. This was added to Materials and Methods.

*Line 331 “most abundant message” typo? mVSG?*

The text was changed to “most abundant mVSG message”.

*Fig 7B is discussed before 7A, switch?*

We switched 7B and C.

*GO term analysis of genes uniquely differentially expressed in metacyclics after CSD1 or CDS2 over expression may help highlight difference in function between the two.*

Thank you for the suggestion. We added the following text “Nonetheless, functional categorization by Gene Ontology (GO) analysis of the set of transcripts down-regulated only in CSD1 metacyclics (179 transcripts) revealed statistically significant enrichment in ribosome biogenesis, rRNA processing, and ribosomal large subunit biogenesis (S1 Table), thus highlighting potential functional differences between CSD1 and CSD2.”

*Legend of Fig 7b, clarify which transcripts this is a summary of (ie seCLIP or RNA-seq, CSD1/2)*

Fig 7B, now Fig 7C, has been replaced with a GO analysis and the figure legend has been expanded.

*The MEME analysis revealed an interesting motif, what percentage the peaks identified by seCLIP have this motif?*

New text “In order to address the RNA sequence specificity of the 308 CSD1 peaks, we used the motif analysis algorithm MEME to search for sequence motifs in a 30-nt window around the RBP binding sites. This analysis retrieved ANACAU in 301 peaks as the most significantly enriched sequence motif (e-value: 1.4^-327^)(Fig 7B).”

*The supplementary table implies this meme analysis was performed on all 289 peaks before normalising, not just high confidence peaks as implied in text. Is this correct? Please clarify*

We apologize for not explaining this clearly. New text “In order to address the RNA sequence specificity of the 308 CSD1 peaks, we used the motif analysis algorithm MEME to search for sequence motifs in a 30-nt window around the RBP binding sites. This analysis retrieved ANACAU in 301 peaks as the most significantly enriched sequence motif (e-value: 1.4^-327^)(Fig 7B). 12 transcripts had multiple peaks and thus, we identified 289 target transcripts for CSD1 (S4 Table).”

*Lines 395-400 discuss results after RNAi depletion of CSD1, please reference these experiments.*

Reference was added.

*Please reference the polyosome experiments in lines 401-406*

Reference was inserted.

*Line 549 – “Filter by quality” please specify this filtering step more clearly, what was the measure of mapping quality? MapQ value?*

The text was modified to “Filter by quality with the default parameters of a quality cut-off value of 20 and 90 percent of bases in sequence that must have quality equal to or higher than cut-off value.”

*Line 439 of the discussion claims “seCLIP-seq results indicated that the binding site of CSD1 and CSD2, as well as their transcript targets were identical”. That is not currently supported as all seCLIP peaks were not found in both cases and this is instead assumed based on the difference in data quality, which is not shown. Could CSD2 instead really just bind a subset of CSD1 targets?*

We agree with the reviewer and changed the text to “seCLIP results indicated that the binding sites of CSD1 and CSD2 are identical and that their transcript targets overlap, either partially or completely.”

*Reviewer #3:*

*Fig 1A - difficult to distinguish between the shading for metacyclic and other*

On further review we realized that there were no other forms and thus removed other.

*figures 4 and 5 - the figures show westerns of expression levels which are fine but do not contain what I promised in the figure title. Could the success of the constructs in causing metacyclic production be included in the figure?*

The figure title was changed to “is essential for inducing mVSG expression” to better reflect what is shown in the Figure.

*line 194 - need a reference for the propensity scale*

Reference was inserted.
